# Evolutionary Relationships of Ljungan Virus Variants Circulating in Multi-Host Systems across Europe

**DOI:** 10.3390/v13071317

**Published:** 2021-07-07

**Authors:** Chiara Rossi, Nicola Zadra, Cristina Fevola, Frauke Ecke, Birger Hörnfeldt, René Kallies, Maria Kazimirova, Magnus Magnusson, Gert E. Olsson, Rainer G. Ulrich, Anne J. Jääskeläinen, Heikki Henttonen, Heidi C. Hauffe

**Affiliations:** 1 Department of Biodiversity and Molecular Ecology, Research and Innovation Centre, Fondazione Edmund Mach, 38098 San Michele all’Adige, TN, Italy; chiara.rossi@fmach.it (C.R.); nicola.zadra@fmach.it (N.Z.); fevolacri@gmail.com (C.F.); 2 Department of Virology, Faculty of Medicine, University of Helsinki, FI-00029 Helsinki, Finland; 3 Department of Wildlife, Fish, and Environmental Studies, Swedish University of Agricultural Sciences, 901 83 Umeå, Sweden; frauke.ecke@slu.se (F.E.); birger.hornfeldt@slu.se (B.H.); magnus.magnusson@skogsstyrelsen.se (M.M.); gert.e.olsson@lansstyrelsen.se (G.E.O.); 4 Department of Environmental Microbiology Working Group Microbial Interaction Ecology, Helmholtz Centre for Environmental Research–UFZ, 04318 Leipzig, Germany; rene.kallies@ufz.de; 5 Slovak Academy of Sciences (SAS), Institute of Zoology, 845 06 Bratislava, Slovakia; Maria.kazimirova@savba.sk; 6 Unit for Nature Conservation, County Administrative Board of Halland County, 301 86 Halmstad, Sweden; 7 Institute of Novel and Emerging Infectious Diseases, Friedrich-Loeffler-Institut, Federal Research Institute for Animal Health, 17493 Greifswald-Insel Riems, Germany; rainer.ulrich@fli.de; 8 HUS Diagnostic Center, HUSLAB, Clinical Microbiology, University of Helsinki and Helsinki University Hospital, FI-00029 Helsinki, Finland; anne.jaaskelainen@helsinki.fi; 9 Wildlife Ecology, Natural Resources Institute Finland (LUKE), FI-00790 Helsinki, Finland; ext.heikki.henttonen@luke.fi

**Keywords:** *Picornaviridae*, *Parechovirus B*, Ljungan virus isolates, small mammals, rodent-borne virus, zoonosis, bank vole

## Abstract

The picornavirus named ‘Ljungan virus’ (LV, species *Parechovirus B*) has been detected in a dozen small mammal species from across Europe, but detailed information on its genetic diversity and host specificity is lacking. Here, we analyze the evolutionary relationships of LV variants circulating in free-living mammal populations by comparing the phylogenetics of the VP1 region (encoding the capsid protein and associated with LV serotype) and the 3D^pol^ region (encoding the RNA polymerase) from 24 LV RNA-positive animals and a fragment of the 5′ untranslated region (UTR) sequence (used for defining strains) in sympatric small mammals. We define three new VP1 genotypes: two in bank voles (*Myodes glareolus*) (genotype 8 from Finland, Sweden, France, and Italy, and genotype 9 from France and Italy) and one in field voles (*Microtus arvalis*) (genotype 7 from Finland). There are several other indications that LV variants are host-specific, at least in parts of their range. Our results suggest that LV evolution is rapid, ongoing and affected by genetic drift, purifying selection, spillover and host evolutionary history. Although recent studies suggest that LV does not have zoonotic potential, its widespread geographical and host distribution in natural populations of well-characterized small mammals could make it useful as a model for studying RNA virus evolution and transmission.

## 1. Introduction

Small mammals, especially mice, rats, voles and bats, are known to be reservoirs and vectors of zoonotic viruses [1,2] many of which are relatively unknown, but whose potential emergence is an increasing burden on socio-economic resources [3,4,5]. The molecular characterization of circulating virus strains and information on their host range and transmission risk can aid the development of highly sensitive diagnostics with a direct effect on public health [6].

Viruses of the family *Picornaviridae* are found in almost all environments and in a great variety of host species, including humans, other mammals, and birds, but also ectotherms such as amphibians [7] and fish [8,9]. They can cause a wide variety of diseases affecting the respiratory and gastrointestinal tract, central nervous system, heart, skeletal muscles and liver. The most studied picornaviruses are those pathogenic to mammals and birds and associated with human and livestock diseases [10,11]. Picornaviruses are known to be mainly species-specific, but the diversity within this family and their hosts is far from being fully delineated. 

One member of this family, Ljungan virus (LV; species *Parechovirus B*), has been the focus of an ongoing discussion on its suggested role in human gestational pathologies, as well as type 1 diabetes [12,13,14,15,16,17,18,19]. LV was first discovered in bank voles (*Myodes glareolus*), and soon after proposed as a rodent-borne zoonotic virus [20]. However, although high LV immunoglobulin G (IgG) seroprevalence (36–38%) has been reported in humans in Finland [21,22], no specific symptoms have been linked to LV infection [18,23,24], and it has been also verified that concurrent LV infections do not appear to influence the clinical picture for disease caused by the rodent-borne Puumala orthohantavirus (PUUV) [23]. LV transmission from rodents to humans has not been confirmed; in fact, on the basis of age-related seroprevalence with a peak in children, Jääskeläinen et al. [22] suggested that LV is not zoonotic, and that LV-reactive antibodies in humans might instead be induced by a human-specific ‘LV-like virus’. To date, LV RNA has been detected in a total of 12 vole, lemming, mouse, shrew and squirrel species collected from nine European countries with a mean RNA prevalence of 15.2% [25]. Thus far, LV has been associated with type 1 diabetes and myocarditis in captive wild voles [26,27,28], especially under stress, as well as gestational pathologies in laboratory mice [29]; however, LV does not appear to influence rodent cycles [30].

The positive single-stranded RNA genome of LV encodes a single polyprotein that is cleaved into 11 proteins with the VP1 region being commonly used to determine LV genotypes [31,32]. However, only eight complete genomes from five VP1 region-based genotypes are known (see also https://talk.ictvonline.org/ictv-reports/ictv_online_report/positive-sense-rna-viruses/w/picornaviridae/693/genus-parechovirus; accessed on 1 May 2021): VP1 genotype 1: isolate 87-012 (virus name Ljungan virus 1) and 174F (unclassified); and genotype 2: isolate 145SL (Ljungan virus 2), 340 (unclassified) and 342 (unclassified). Both genotypes 1 and 2 were originally isolated from bank voles captured in Sweden and passaged in baby hamster kidney (BHK)-21 cells injected into laboratory mice [20,32]. Genotype 3 includes isolate M1146 (Ljungan virus 3) from the montane vole (*Microtus montanus*) [33]; and genotype 4, the isolate 64-7855 (Ljungan virus 4) from the southern red-backed vole (*Myodes gapperi*) [31], both native rodents in the USA. Finally, genotype 5 includes isolate Fuz1 (Ljungan virus 5), isolated from wild birds in Japan [34]. In addition, putative genotype 6 from RtMrut-PicoV/JL2014-2 (Ljungan virus 6), represents a new candidate member of the *Parechovirus* genus, sequenced from the northern red-backed vole (*Myodes rutilus*) in China [35].

More detailed molecular information about LV, from new hosts and locations, is necessary to better understand its genetic diversity, host specificity and zoonotic potential [13,22,25,32,36]. Therefore, the purpose of this study was to analyze the evolutionary relationships of LV variants circulating in multi-host small mammal communities across Europe and to discuss the implications of the results for host range and transmission risk.

## 2. Materials and Methods

### 2.1. Sample Collection

Liver samples were obtained from small mammal species (nomenclature following Integrated Taxonomic Information System; https://www.itis.gov/ accessed on 1 May 2021) trapped during the EU FP7 Emerging Diseases in a Changing European Environment project (EDENext; http://www.edenext.eu/ accessed on 1 April 2019) and additional national projects, and described in detail in [25] and Supplementary Table S1.

We made several attempts to sequence entire genomes from LV RNA extracted from wild small mammals (following [32] in collaboration with the European Virus Archive), including RNA enrichment but excluding cell culture passaging, but were unsuccessful. Hence, we concluded that the viral load in these samples (and therefore, total LV RNA) was too low to proceed to whole genome sequences. For this reason, to complete our phylogenetic studies, we decided to sequence two genetic markers: (a) a 393 nucleotide (nt) fragment of the VP1 region; and (b) a 471 nt fragment of the 3D^pol^ region encoding the RNA polymerase. The VP1 genotype was chosen because it corresponds to LV serotype; since genotype/serotype can induce different responses in the host, its classification is important for understanding virus ecology. Instead, the 3D^pol^ region was selected because it is used extensively in phylogenetic studies for members of the *Picornaviridae* family [37]. In addition, we used 137 nt sequences of the 5′-untranslated region (UTR) generated by [25], in order to investigate host specificities of these LV 5′-UTR haplotypes in small mammals, as this fragment is often used for detecting strains within genera of picornaviruses [38,39,40,41].

### 2.2. RNA Extraction, Reverse Transcription-Polymerase Chain Reaction (RT-PCR) and Sequencing

Total RNA was extracted from LV PCR-positive liver samples identified in [25], as described in [42]. Single-step and nested RT-PCRs were performed using primers targeting the 3D^pol^ and VP1 regions (see Supplementary Table S2). Initially, we designed primers based on the deposited genomes of LV and human parechovirus (HPeV) and obtained a low number of sequences for both markers. Therefore, using these sequences and those available in public databases, we designed additional primers (see Supplementary Table S2), which we used in combination with the original ones in nested RT-PCRs to obtain additional sequences. RT-PCRs were performed with the OneStep RT-PCR kit (QIAGEN, Hilden, Germany) on a Veriti^®^ Thermal Cycler (Applied Biosystems, Foster City, CA, USA) using 4 µL of total RNA following the manufacturer’s instructions with the following modification: a touch-down was carried out for both genes of interest (VP1: 60 °C–54 °C × 7 cycles; 54 °C × 43 cycles; 3D^pol^: 60 °C–52 °C × 9 cycles; 52 °C × 41 cycles). Nested RT-PCRs were performed with 2 µL of cDNA on the same thermal cycler using the AmpliTaq Gold^®^ 360 PCR Master Mix (Thermo Fisher Scientific, Waltham, MA, USA) following the manufacturer’s instructions with the following modification: a touch-down was carried out as described above. PCR products were purified with the PCR Purification Combo Kit (Invitrogen, Carlsbad, CA, USA) and sequenced by dideoxy chain-termination protocol on an ABI PRISM 3730xl Genetic Analyzer (Applied Biosystems) using the BigDye Terminator cycle sequencing kit (Perkin Elmer, Applied Biosystems Division, Foster City, CA, USA). Sequences were edited using Sequencher DNA sequence analysis software (version 4.7, Gene Codes, Ann Arbor, MI, USA) and confirmed using the Basic Alignment Search Tool (BLAST^®^) (version BLASTN 2.12.0+) analysis available in the National Center for Biotechnology Information (NCBI) [43].

### 2.3. Genotyping Using the VP1 Region

According to the criteria for genotyping enteroviruses (EVs; family *Picornaviridae*) [31,32,44], a genotype is identified based on the VP1 region when, compared with already known genotypes, nucleotide sequence identity of this region is less than 75% and amino acid sequence identity of the encoded protein is less than 88%. Therefore, in order to identify genotypes among our sequences, the VP1 genetic distances were calculated in MEGA 5.2 [45], with the p-distance method as the model of nucleotide substitution. We also included LV VP1 nucleotide and amino acid sequences available in GenBank: 87-012 (GenBank acc. no.: AF327920.2), 174F (AF327921.2), 145SL (AF327922.2), 340 (KR045607.1), 342 (KR045608.1), M1146 (AF538689.1), 64-7855 (EU854568.1), FUZ1 (LC133331.1) and RtMrut-PicoV/JL2014-2 (KY432929).

To support the genotyping, phylogenetic relationships among VP1 sequences were reconstructed with a maximum likelihood (ML) algorithm in phyML ver. 3.1 [46] using the approximate log likelihood ratio test (aLRT) to evaluate node supports and Bayesian inference (BI) in Mr. Bayes ver. 3.1.2 [47], run for 10 million of generations, and sampled every 100th generation, with a burn-in of 50%. The best-fit model was selected with jModeltest2 [48] on the CIPRES Science Gateway [49] under the Akaike Information Criterion. Trees were visualized and modified with Fig Tree v. 1.3.1 [50].

### 2.4. Analysis of Potential Selection on VP1 Region

VP1 region encodes for a structural protein that interacts with the host immune system and is potentially subject to selection. Therefore, using VP1 sequences generated both here and previously (see GenBank acc. no. listed above) from various host species and geographic origins, we performed an analysis of codon positions under selective pressure by comparing results from three methods. Given the lack of previous knowledge on how LV evolves, we applied two methods which detect sites under selection according to the dN-dS ratio (ω): fixed effects likelihood (FEL) [50], which assumes a constant selective pressure along the history of the virus at a particular site, and mixed effects model of evolution (MEME) [51], which instead detects episodic evolutionary processes (http://www.datamonkey.org) accessed on 2 July 2019 [52]. We integrated these approaches based on ω ratio with the analysis of the changes in the amino acid properties when a substitution occurs. We analyzed selection on 31 physicochemical amino acid properties of VP1 using TreeSAAP 3.2 [53] accessed on 1 August 2019 by applying a windows analysis approach with a width of 15 residues. In addition, we applied this method to FEL and MEME, as the rate of synonymous substitutions tends to be higher than the rate of nonsynonymous substitutions even when a site is effectively under positive selection [54], to avoid being too conservative [53]. The TreeSAAP categories 1 to 8 indicate the type of selection acting on the fragment windows: lower magnitude (categories 1–3) with a Z-score > 3.09 indicate stabilizing (purifying) selection, whereas the higher categories (6-8) with a Z-score > 3.09 indicate positive (destabilizing) selection [55].

### 2.5. Phylogenetic Analysis of the 3D^pol^ Region

The genetic distance and phylogenetic relationships of nucleotide/amino acid sequences of 3D^pol^ region were calculated as described above for VP1. We also included here the known LV strains available in public databases. In addition, we added Falcon/HA18_080/2014/HUN (KY645497), a closely related parechovirus isolated from birds of prey in Hungary, and SEBV-1 (NC_021482), a rodent-borne parechovirus isolated in Central Africa. Two HPeV strains HPeV2 (AJ005695.1) and HPeV4 (AB433629.1), chosen from the available HPeV strains as representative of the genetic diversity of *Parechovirus A*, were included as outgroups. A pairwise comparison of the 3D^pol^ nucleotide sequences was performed within and between the clusters resulting from the phylogenetic analysis.

### 2.6. Network Analysis Using the 5′-UTR

**Figure 1:**
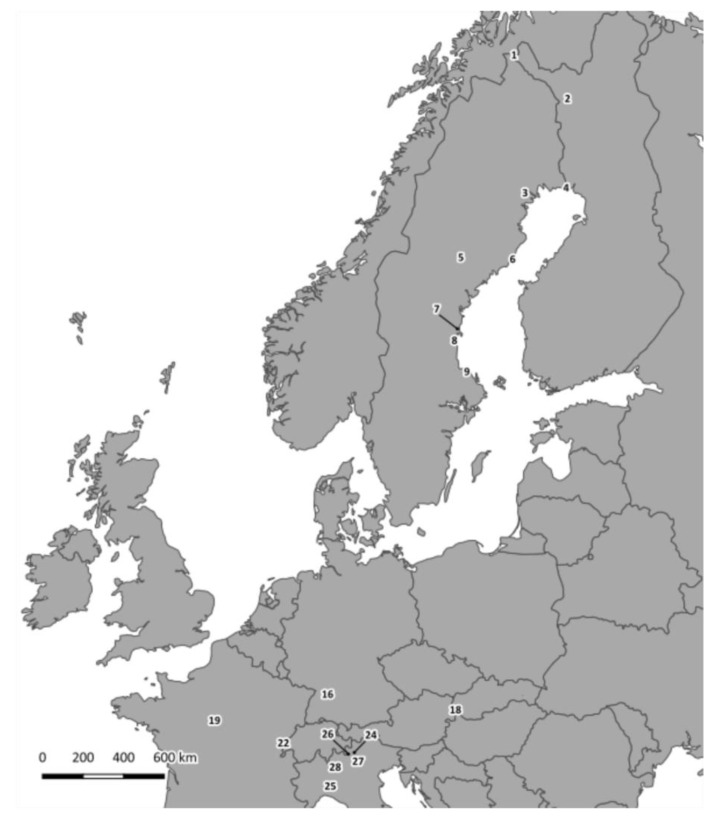
Map of sampling sites in Europe where LV sequences analyzed in this paper were detected. Numbers match those in Table S1. Number: nearest location, country (acronym) are: 1: Kilpisjärvi, Finland (KJ); 2: Pallasjärvi, Finland (PJ); 3: Harads, Sweden (HA); 4: Haparanda, Sweden (HP); 5: Fredrika, Sweden (FE); 6: Umeå coast, Sweden (UM); 7: Gnarp, Sweden (GN); 8: Enånger, Sweden (EN); 9: Tierp, Sweden (TI); 16: Weissach, Germany (WE); 18: Fugelka, Slovakia (FU); 19: La Venotiere, France (LA); 22: Mignovillard, France (MI); 24: Brescia, Italy (BS); 25: Pavia, Italy (PV); 26: Sondrio, Italy (SO); 27: Trento, Italy (TN); 28: Lecco, Italy (LC). Sites with numbers 10–15, 17, 20, 21, 23 and 29 are not shown here, as no LV RNA was detected in animals from these sites.

To minimize the homoplasy masking phylogenetic patterns in these short, but variable sequences, noted by [25], we performed a network analysis of a subset of the 5′-UTR haplotypes (137 nt) from geographical areas where sequences from multi-species small mammal communities were available, i.e., northern Italy (TN, SO, BS, LC and PV sites; *N* = 40 sequences, five species including Cricetidae/Arvicolinae, Muridae, Sciuridae and Soricidae) and Finnish Lapland (PJ and KJ sites; *N* = 18 sequences, five species of Arvicolinae) (see Figure 1 for sampling sites). The list of samples used can be found in Supplementary Table S3. The two networks were generated using TCS [56] and visualized with PopArt (http://popart.otago.ac.nz) accessed on 3 April 2020.

## 3. Results

### 3.1. Genotyping Using the VP1 Region

**Figure 2:**
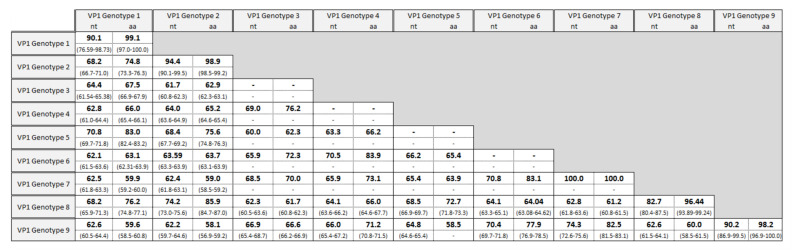
VP1 genotypes identified from 393 nt fragments in wild rodents from this study and available in public databases and mean values of inter- and intra-genotype identity (and range) of nt and amino acid sequences ^1^. ^1^ Genotype 1: 87-012 (GenBank acc. no.: AF327920.2) and 174F (AF327921.2); genotype 2: 145SL (AF327922.2), 340 (KR045607.1) and 342 (KR045608.1); genotype 3: M1146 (AF538689.1); genotype 4: 64-7855 (EU854568.1); genotype 5: FUZ1 (LC133331.1); genotype 6: RtMrut-PicoV/JL2014-2 (KY432929)): (see Table 1). For genotypes 3, 4, 5 and 6, intra-genotype values are lacking because genotypes were represented by a single sequence.

A total of 90 samples from 12 mammal species and nine countries were analyzed in this study (Figure 1). We were able to amplify partial VP1 sequences from 21 bank vole individuals and three field vole individuals (*Microtus agrestis*; Table 1). Single LV variants were found in each vole. Based on a 75% nt sequence identity and 88% amino acid sequence identity (1 minus p-distances), nine VP1 genotypes were identified (Figure 2). Nt sequence identities ranged from 59.7 to 75.6% (inter-genotype identity) and 76.6 to 100% (intra-genotype identity), while amino acid sequence identities ranged from 56.9 to 87.5% and 93.9 to 100%, respectively. Although the interval between the highest intra-genotype and lowest inter-genotype nucleotide sequence identity is narrow (75.64 vs. 76.59%), the gap between the two groups defined by amino acid sequence similarity is more clear-cut (87.53 vs. 93.89%), and no value of inter-genotype amino acid sequence identity exceeds the threshold of 88%.

A total of 11 VP1 sequences, nine from Sweden (sites: HP, FE, UM, GN, TI), one from Germany (WE) and one from Slovakia (FU) coincide with previously reported VP1 genotype 1 from Sweden (Table 1) [20]. Only one sample, collected in Sweden (EN), matched the previously noted genotype 2 (also from Sweden) [20,32]. None of our sequences matched genotypes 3 or 4 (originally identified in voles from the USA) [31,33], genotype 5 (from birds in Japan) [34] or genotype 6 (from northern red-backed vole in China) [35]. In addition, a new VP1 genotype, genotype 7, was shared by three field voles captured in Finland (site PJ). New genotype 8 was found in four bank voles, one each from Finland (PJ), Sweden (UM), Italy (TN) and France (MI). New genotype 9 was identified in bank voles sampled in Italy (one from BS and two from SO) and France (two from LA).

Several pairs of sequences approach the 75 and 88% genotype cut-offs for nt and amino acid sequence identity: two variants of genotype 2 have similar nt sequences to two variants of new genotype 8 (genotype 2: 13-Mg-EN-SE vs. genotype 8: 2-Mg-PJ-FI: 75.6%, and genotype 2: strain 340 (Genbank acc. no. KR045607.1) vs. genotype 8: 18-Mg-MI-FR: 75.3%). The three variants of genotype 7 (1-Ma-PJ-FI, 2-Ma-PJ-FI and 3-Ma-PJ-FI) are equally similar to one variant of new genotype 9 (22-Mg-SO-IT; 75.6%). In addition, amino acid sequence identity values between genotypes 2 and 8 (85.5%, 86.3% for the two comparisons above, respectively) and genotypes 7 and 9 (83.1%) are lower than 88%. Therefore, the genotype pairs 2/8 and 7/9 could be considered sister genotypes.

### 3.2. Phylogeny of the VP1 Region

**Figure 3:**
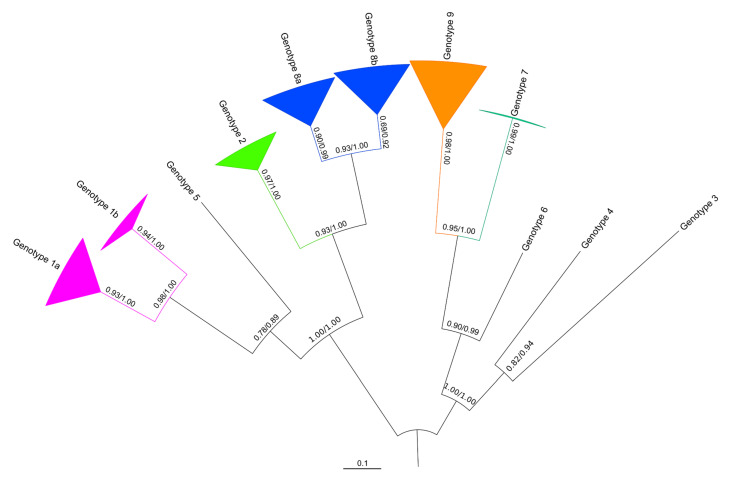
Phylogenetic tree of VP1 nt sequences generated for LV strains from the wild rodents listed in Table 1. Support values are found above or next to each node: approximate likelihood ratio test of the maximum likelihood (aLRT)/posterior probabilities of the Bayesian reconstruction (BPP). Genotype sequences with over 85% identity were collapsed.

Both ML and BI algorithms provided an identical tree topology of VP1 nucleotide sequences with highly supported nodes (Figure 3), confirming the genotyping based on p-distances as described in the previous paragraph. The topology revealed a star-like tree, with long basal branches. Both algorithms generated three clusters including genotypes 1 and 5, 2 and 8, and 7 and 9. Genotypes 1 and 8 also show intra-genotype bifurcation, with differentiation between sequences from northern (VP1 genotype 1a; see also Table 1) and central (VP1 genotype 1b) Europe. Similarly, genotype 8 shows differentiation between northern (VP1 genotype 8a) and southern (VP1 genotype 8b) variants. 

### 3.3. Molecular Analysis of VP1 Sequences

**Figure 4:**
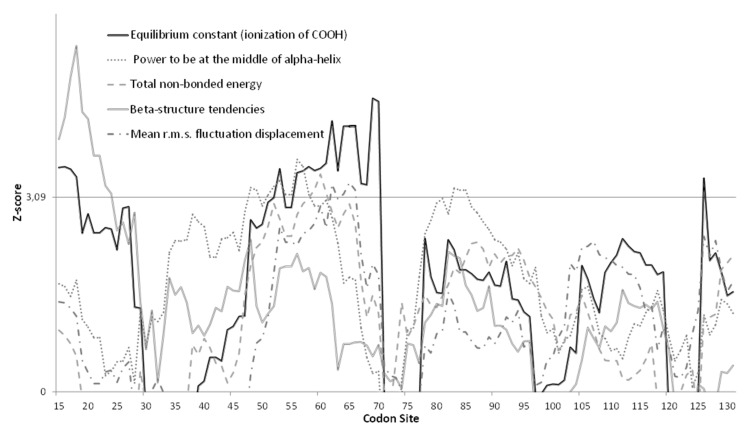
Results of the TreeSAAP sliding analysis with a window width of 15 residues of the VP1 amino acid sequences (see also Table 1). The *X*-axis indicates the last codon of the window. The *Y*-axis corresponds to the Z-score values for the five properties that reach the threshold of 3.09 [54]. Biochemical properties affected by stabilizing selection (categories 1–3) are shown in grey with dashed lines; the one affected by destabilizing selection (categories 6–8) in black.

Neither FEL nor MEME detected sites under positive selection. TreeSAAP only identified one property under positive destabilizing selection (*Equilibrium constant: ionization of COOH*; category 8) with a Z-score higher than the threshold 3.09, and four properties under conservative selection (*Beta-structure tendencies, Mean root square fluctuation displacement, Total non-bonded energy*; category 1, *Power to be at the middle of alpha-helix*; category 2) with a statistical support (Z-score) above the threshold of 3.09 (Figure 4). Residues affected by destabilizing selection and conservative selection are shown in Supplementary File S1.

### 3.4. Phylogeny of 3D^pol^ Region

**Figure 5:**
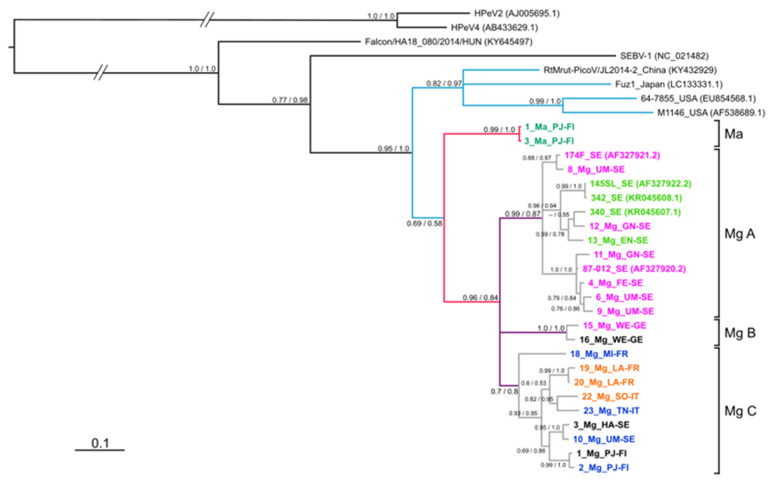
Phylogenetic tree of the 3D^pol^ nt sequences generated from the field vole (Ma) and bank vole (Mg) LV RT-PCR-positive samples listed in Table 1, with additional sequences available in GenBank, including two HPeV sequences as outgroups: HPeV2 (AJ005695.1) and HPeV4 (AB433629.1). The three Mg clusters are named A, B and C. Samples are highlighted according to VP1 genotypes (see Table 1; Figure 3): VP1 genotype 1 (pink), 2 (light green), 7 (dark green), 8 (blue) and 9 (orange) (black: VP1 variants not available). Branch colors highlight: bank vole (Mg)-associated clusters A, B and C (purple); relationship between bank vole and field vole clusters (red); relationships between European and non-European sequences LV 64-7855, LV M1146, LV Fuz1 and RtMrut-PicoV/JL2014-2 (light blue). Support values to the left of each node indicate the approximate likelihood ratio test of the maximum likelihood (aLRT)/posterior probabilities of the Bayesian reconstruction (BPP). The length of the bar indicates the phylogenetic distance.

We sequenced 3D^pol^ fragments of LV strains from 18 bank voles and two field voles (Table 1), with each individual carrying a single LV variant. Both phylogenetic methods ML and BI provided the same cladogram (Figure 5). All known LV strains clustered separately from closely related bird Falcon/HA18_080/2014/HUN (KY645497) and rodent SEBV-1 (NC_021482) parechovirus sequences. The four LV strains from Japan (Fuz1), China (RtMrut-PicoV/JL2014-2) and the USA (64-7855 and M11465) formed a cluster well-separated from that of all the European sequences with a high level of branch support (aLRT/BPP: 0.95/1.0). Within the European sequences, the bank vole-associated LV strains formed a monophyletic group with respect to the two field vole-associated sequences with high support (0.96/0.84; aLRT/BPP).

There were three main branches within the clade containing the bank vole sequences (labelled subgroups Mg A, B and C in Figure 5). The subgroups were all equally related to each other and were well-supported (aLRT/BPP: 0.99/0.87, 1.00/1.00, 0.7/0.8). Subgroup Mg A contained our sequences from Sweden, as well as previously published sequences belonging to VP1 genotypes 1 and 2 (Table 1). Subgroup Mg B is characterized by two variants from Germany, with one from genotype 1 (VP1 of the second variant was not generated; Table 1). Subgroup Mg C is characterized by sequences from bank voles from different sites across Europe: Finland (PJ), Sweden (HA and UM), Italy (TN and SO), and France (LV and MI). The variants belonging to this subgroup had sequences matching VP1 genotypes 8 and 9 (Table 1).

### 3.5. Molecular Analysis of 3D^pol^ Region

**Figure 6:**
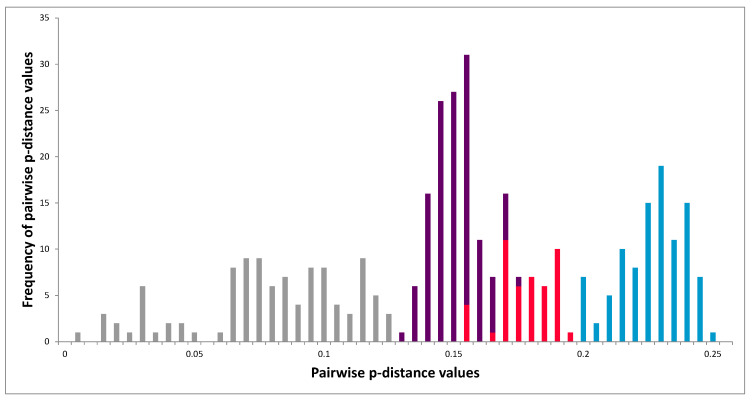
Frequency of the pairwise p-distance values of the 3D^pol^ nucleotide sequences generated by MEGA. Bar colors correspond to sequences and clusters as in Figure 5.

Pairwise comparisons of the 3D^pol^ nt sequences revealed that the overall mean divergence was 0.150 with a maximum value of 0.248 and a minimum value of 0.002 (Figure 6). Among the bank vole- and field vole-associated clusters, these values varied from 0.153 to 0.193, while between bank vole clusters, the intra-cluster values ranged from 0.002 to 0.121 and inter-cluster values from to 0.130 to 0.176 (grey bars in Figure 6). The mean divergence within subgroups Mg A and Mg C was comparable (0.79 and 0.77, respectively), as was the range of variability (Mg A: 0.002–0.119; Mg C: 0.013–0.121). Subgroup Mg B has the lowest divergence (0.034), but this cluster only has two members, both from the same sampling site (Germany: WE).

### 3.6. Networks of 5′-UTR Haplotypes

**Figure 7:**
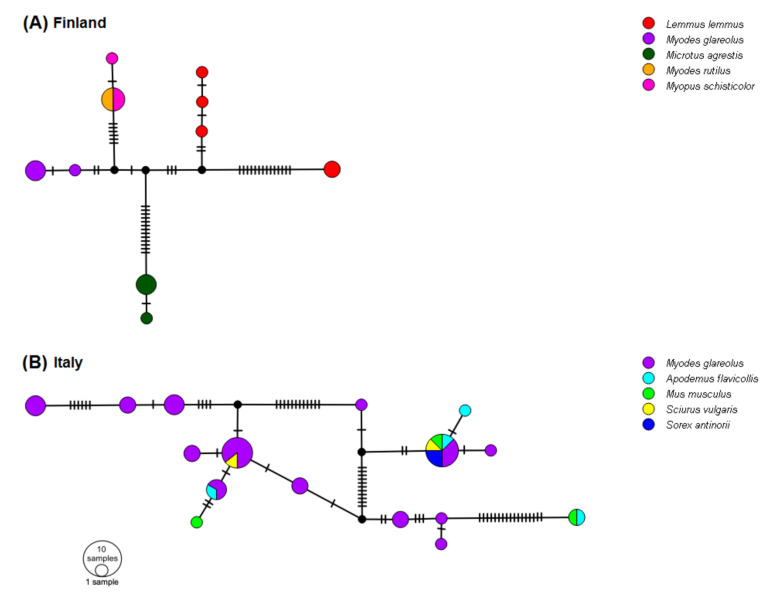
Network of 5′-UTR sequences from small mammal communities generated by POPART. (**A**) Small mammal community in Finland, sampling sites KJ and PJ. (**B**) Small mammal community in Italy, sampling sites BS, LC, PV, SO and TN.

The TCS network of Finnish haplotypes had a central node with five clusters including variants associated with either bank vole, field vole, northern red-backed vole or wood lemming (*Myopus schisticolor*); and two clusters were associated with variants for Norway lemming (*Lemmus lemmus*; Figure 7A). For Italy, the majority of 5′-UTR haplotypes originated from bank voles and the network showed no particular pattern according to host species or sampling site (Figure 7B).

## 4. Discussion

### 4.1. LV Phylogeny and Evolution

The zoonotic potential of mammal-borne viruses has been brought to the forefront during the current pandemic. Estimating this potential requires knowledge of genetic diversity and host specificity. This is the first study to investigate the genetic diversity of LV at multiple molecular markers from various wild small mammal hosts across its geographical range. Using LV-positive samples from [25], we sequenced two additional markers (VP1 and 3D^pol^) in order to assign variants to known or new genotypes, and to reconstruct how these variants are phylogenetically related and distributed across Europe. Host specificity is investigated through a phylogenetic analysis of the 5′-UTR haplotypes in two small mammal communities.

Members of the *Picornaviridae* family are known to have high sequence variability in the part of the genome encoding the capsid protein VP1, responsible for the host immune response [57,58,59], as also shown for LV [31]. Here we generated VP1 sequences to classify new LV VP1 variants. Using standard cut offs for nt (75%) and amino acid (88%) sequence identity, we confirmed the presence of genotypes 1 and 2 in Swedish bank voles, as noted by previous authors [20,32], as well as in Germany and Slovakia. We also confirmed the lack of genotypes 3 and 4 (USA; voles), 5 (Japan; birds), and genotype 6 (China; northern red-backed vole) in Europe (Figure 3). We named two other new VP1 genotypes in our European bank vole samples as genotype 8 (from Finland, Sweden, France, and Italy) and genotype 9 (from France and Italy). We also noted genotype 7 in Finland that was only found in field voles.

The phylogeny of 3D^pol^ variants (Figure 5) mirrors the VP1 genotype distribution with sequences from China, Japan and the USA clustering outside the European samples, while within the European samples, a field vole LV cluster and three closely related bank vole-associated LV lineages are present. However, the phylogeny of VP1 sequences is more complex. For example, genotypes 1 and 5 are closely related in the VP1 tree, but paraphyletic in the 3D^pol^ tree (Figure 3) and not geographically sympatric; genotype 7 (from field vole) and 9 (from bank vole) are related in the VP1 tree (Figure 2), but found in genetically distant clusters defined by host species in the 3D^pol^ tree (Figure 5). This discordance is probably due to evolutionary processes occurring in the VP1 region. Firstly, in the VP1 sequences presented here, the nucleotide sequence identity is higher than the amino acid sequence identity (59.74 and 56.92%, respectively), suggesting that there is a higher frequency of non-synonymous substitutions compared to synonymous ones between genotypes than within genotypes. Such a ratio is required to maximize the divergence of biochemical properties of amino acid residues of the capsid proteins; this variation modifies epitopes in an attempt to circumvent host immune responses. If a constant mutation rate, generally high in picornaviruses [59,60] is assumed, with frequent production of deleterious or lethal mutations, as noted for RNA viruses [61], the phylogenetic similarity of some pairs of VP1 genotypes can be explained by purifying selection, which is recurrent along the LV genome as observed by [32]. This is confirmed by our TreeSAAP analysis (Figure 4), which showed that where destabilizing positive selection operates, purifying selection appears to act to maintain the functionality of the protein.

Several LV genotypes show signs of continuing evolution and divergence. The star-like phylogeny of VP1 and the unresolved phylogeny of bank vole-derived LV 3D^pol^ sequences suggest that LV lineages occur independently rather than being derived from each other. In addition, genotype 1 was identified in many bank vole samples from Sweden, but also from two samples in Germany and Slovakia: while the mean nt and amino acid sequence identities of this genotype are 90.12 and 99.11%, respectively (Figure 3), the two central European samples have a much lower nt sequence identity with respect to the mean value of identity of VP1 genotype 1: 79.42% (15_Mg_WE-GE) and 80.60% for (17_Mg_FU-SK), close to the cutoff threshold (75%) for defining a new genotype. However, the amino acid sequence identity within the genotype is 97.76% for both the samples, suggesting that the nucleotide sequence differences are due to genetic drift in geographic isolation. Genetic drift due to geographical distance between genotype distributions could also explain the discrepancy between a low value of nucleotide sequence identity (82.52%), and relatively high amino acid sequence identity (96.41%) for genotype 8. Interestingly, the alignment of VP1 amino acid sequences (see Supplementary File S1) indicates highly variable regions, which might be conceived as putative epitopes that characterize each genotype serologically [31,62]. Because some pairs of genotypes (genotypes 1 and 5; genotypes 2 and 8) have similar amino acids in this region, they may also be serologically similar.

Recombination is known to generate discordant cladograms when different segments of viral genomes are analyzed [31,63]. The occurrence of recombination between variants is frequent in picornaviruses [64] including those in the genus *Parechovirus* [37,65,66,67,68]. In addition, the sequences flanking the capsid-encoding region are recognized as a breaking point in HPeV genome [69,70,71]. Recombination has already been hypothesized in a previous phylogenetic analysis of LV [31]. Here, different VP1 genotypes and 3D^pol^ clusters present in the same individual also suggest recombination event(s) in LV evolution. For example, in bank vole A cluster (Mg A; Figure 5), there were animals with both genotypes 1 and 2 (Table 1); within the Mg C cluster, both genotypes 8 and 9 were detected. Since genotype 8 is rather widespread, and genotype 9 is restricted to southern Europe, recombination may have occurred in the latter region. However, we did not find recombinants between genotype 1/Mg A and genotype 8/Mg C, which were both found in Umeå; this absence may indicate incompatibilities of certain recombinants, or alternatively, small sample size, or recent contact between the two groups.

### 4.2. Host Specificity

Knowledge of the host range of a pathogen and monitoring or predicting processes of adaptation to new hosts is an important issue in molecular epidemiology, also because emerging pathogens are often characterized by a shift in host range or a spillover into other hosts. Although many studies regarding LV focused only on bank voles [20,26,27] and LV has been shown to be common in wild bank vole populations [25], it has also been found in 11 other hosts [25,72,73]. Thus, additional rodent species, particularly voles in the Arvicolinae subfamily, could play a role in the circulation of LV in multi-host small mammal systems.

There are several indications that LV variants are host-specific. The Finnish network of 5′-UTR sequences (Figure 7A) suggests several species-specific haplotypes in field voles, Norwegian lemmings and bank voles. The 5′-UTR tree in Figure 7A and the monophyly of the bank vole variants in the 3D^pol^ tree (Figure 5) indicate that there may be some bank vole-specific LV variants. However, the case for host-specific LV haplotypes is strongest for field voles, since only field voles carried VP1 genotype 7 (Table 1) and certain 3D^pol^ variants, which were phylogenetically distant from other clusters (Figure 5), although pairwise p-distances (Figure 6) indicate that differentiation between host-specific haplotypes is not yet complete. The possibility of lemming-specific haplotypes indicated in Figure 7A is strengthened by the result that one haplotype is found in site PJ, but also in KJ, where bank voles are not present [74], hence the maintenance of this haplotype cannot be due to recent spillover, although we cannot exclude the chance that LV presence in this population was initiated by spillover from bank voles in the more distant past. The fact that we were not able to obtain additional sequences of VP1 and 3D^pol^ from other species including humans, with the exception of bank vole or field vole, despite considerable effort by several laboratories using various direct and indirect techniques [24], may also be indicative of host-specific variants that could not be amplified by the primer pairs used here due to substantial divergence, even though the primers were degenerated. This “phylogenetic distance effect” [75] has been noted as especially relevant for amplifying VP1 sequences [76]. Forbes et al. [36] attributed the apparent lack of LV RNA in seropositive field voles as a consequence of events of spillover from sympatric bank voles, but here our findings demonstrate that field vole-specific variants could be a more consistent explanation.

Interestingly, we observed co-presence of two LV clusters in northern Sweden (sites: HA, UM), where genotype 1/3D^pol^ Mg A and genotype 8/3D^pol^ Mg C both occur; north and south of this area, only one of the two occurs, respectively. Since a contact zone between two geographic lineages of PUUV, a bank vole-borne virus, occurs ca. 200 km south of the UM area [77], the distribution of LV clusters may reflect the historical colonization of Fennoscandia by the bank vole, along both northern and southern routes, as shown by the distribution of mitochondrial DNA lineages [78]. However, at the European level, the rapid evolution and wide host range of LV might not allow the comparison of host-virus phylogenies, as observed for PUUV, even though this virus has a single rodent host species [79,80].

In northern Italy, the haplotype shared by one individual of yellow-necked mouse (TN) and one individual of house mouse (*Mus musculus*) (BS), divergent from other 5′-UTR sequences by 17 mutations, may represent a murid-associated lineage (Figure 7B). However, in general, the association of 5′-UTR haplotypes and host species was not observed in this small mammal community (Figure 7B), even in endemic species such as the Valais shrew [81], suggesting that LV spillover from a reservoir host such as the bank vole to other species is possible, a common phenomenon in RNA viruses [61]. Salisbury et al. [73] also observed in the UK that the bank vole, field vole, house mouse and wood mouse (*Apodemus sylvaticus*) share some of the same LV variants. Even in Finland, the northern red-backed vole and wood lemming shared haplotypes (Figure 7A). Forbes et al. [36] hypothesized a major role of bank vole in maintaining a high prevalence of LV in sympatric species. Bank vole may also play a major role in our study area, since this host species is present in all the Italian sites analyzed here, and populations are known to be genetically connected across this geographical area [82,83]. The reason for the contrasting patterns of specificity in Finnish and Italian rodent communities is unclear. Little is known regarding the transmission of LV among host individuals, although an oral-fecal route has previously been suggested [20]. Therefore, we suggest that the spread of LV might be connected with host interactions and dispersal, as well as abiotic factors, as previously noted in [25]. Larger sample sizes of alternative potential host species, including birds, are needed in order to confirm their role as reservoirs and in LV transmission.

## 5. Conclusions

LV is a widespread, rapidly evolving RNA virus present in numerous small mammal species across nine European countries. The distribution of genetic variants from three different segments of the genome (VP1, 3D^pol^ and 5′-UTR) suggest that LV evolution is ongoing and affected by genetic drift, purifying selection, recombination events, spillover and host evolutionary history. Some host specificity also appears to have evolved or is evolving. Although recent research has indicated that LV is not associated with human disease and is considered to have a low zoonotic potential, its widespread geographical and host distribution in natural populations of well-characterized small mammals could make it useful as a model for studying RNA virus evolution and transmission.

## Figures and Tables

**Table 1:** List of LV RT-PCR-positive rodent samples from Europe with sequences available for analysis ^1^, including geographical origin, VP1 genotype and 3D^pol^ phylogenetic subgroup.

Sample Number ^2^	Species	Site ^3^	Country	VP1 Genotype ^4^	3D^pol^ Subgroup ^5^
1-Ma-PJ-FI ^6^	*Microtus agrestis*	PJ	Finland	7	Ma
2-Ma-PJ-FI ^6^	*Microtus agrestis*	PJ	Finland	7	ND ^7^
3-Ma-PJ-FI ^6^	*Microtus agrestis*	PJ	Finland	7	Ma
1-Mg-PJ-FI ^6^	*Myodes glareolus*	PJ	Finland	ND	Mg C
2-Mg-PJ-FI ^6^	*Myodes glareolus*	PJ	Finland	8	Mg C
3-Mg-HA-SE	*Myodes glareolus*	HA	Sweden	ND	Mg C
4-Mg-HP-SE	*Myodes glareolus*	HP	Sweden	1	Mg A
5-Mg-FE-SE	*Myodes glareolus*	FE	Sweden	1	ND
6-Mg-UM-SE	*Myodes glareolus*	UM	Sweden	1	Mg A
7-Mg-UM-SE	*Myodes glareolus*	UM	Sweden	1	ND
8-Mg-UM-SE	*Myodes glareolus*	UM	Sweden	1	Mg A
9-Mg-UM-SE	*Myodes glareolus*	UM	Sweden	1	Mg A
10-Mg-UM-SE	*Myodes glareolus*	UM	Sweden	8	Mg C
11-Mg-GN-SE	*Myodes glareolus*	GN	Sweden	1	Mg A
12-Mg-GN-SE	*Myodes glareolus*	GN	Sweden	1	Mg A
13-Mg-EN-SE	*Myodes glareolus*	EN	Sweden	2	Mg A
14-Mg-TI-SE	*Myodes glareolus*	TI	Sweden	1	ND
15-Mg-WE-DE	*Myodes glareolus*	WE	Germany	1	Mg B
16-Mg-WE-DE	*Myodes glareolus*	WE	Germany	ND	Mg B
17-Mg-FU-SK	*Myodes glareolus*	FU	Slovakia	1	ND
18-Mg-MI-FR	*Myodes glareolus*	MI	France	8	Mg C
19-Mg-LA-FR	*Myodes glareolus*	LA	France	9	Mg C
20-Mg-LA-FR	*Myodes glareolus*	LA	France	9	Mg C
21-Mg-SO-IT ^6^	*Myodes glareolus*	SO	Italy	9	ND
22-Mg-SO-IT ^6^	*Myodes glareolus*	SO	Italy	9	Mg C
23-Mg-TN-IT ^6^	*Myodes glareolus*	TN	Italy	8	Mg C
24-Mg-BS-IT ^6^	*Myodes glareolus*	BS	Italy	9	ND

^1^ For VP1 and 3D^pol^ analysis; additional samples for 5′-UTR analysis listed in Supplementary Table S3. ^2^ Abbreviations (also used in subsequent Figures) determined by: ID number, species, sampling site (see Figure 1), country code. Ma: *Microtus agrestis*; Mg: *Myodes glareolus*. ^3^ Listed in order from north to south; for abbreviations see footnote Figure 1. ^4^ See also Figure 3; genotypes 1 and 2 previously noted in [20,32]. ^5^ A–C represent different Mg clusters. See also Figure 4. ^6^ Samples also used to generate 5′-UTR sequences for network analysis. ^7^ ND: not determined, i.e., a sequence could not be generated from this sample for this locus.

## Data Availability

All data from this study are available within this manuscript and its Supplementary Material.

## Supplementary Materials

The following supplementary tables and txt files are available online at https://www.mdpi.com/article/10.3390/v13071317/s1: Table S1: Site of origin for samples analyzed for LV VP1 and 3D^pol^ regions. Table S2: List of primers used to amplify LV 3D^pol^ and VP1 region. Table S3: Samples used in the network analysis based on 5′-UTR sequences, including country and site of origin, host species and family/subfamily (subf.). File S1: VP1 amino acid sequence alignment.
