# Supplementary material for: Evolutionary Relationships of Ljungan Virus Variants Circulating in Multi-Host Systems across Europe
Source: Viruses. 2021 Jul 7;13(7):1317. doi: 10.3390/v13071317 (PMC8310206; doi:10.3390/v13071317)
Supplement: Supplementary file 1 [file viruses-13-01317-s001.zip › Supplementary Table S1-S2-S3.pdf]

Supplementary Table S1. Site of origin for samples were analysed for LV VP1 and 3D^{pol} regions. See also Figure 1. For each site the nearest location, country of origin, host species, trapping year and geographical coordinates are shown.

Sampling site	Nearest location (acronym)	Country	Host species (n°individuals)	Trapping year	Latitude/Longitude
**1**	Kilpisjärvi (KJ)	Finland	*Lemmus lemmus* (3)	2010-2011	69°02'34"N, 20°48'19"E
**2**	Pallasjärvi (PJ)	Finland	*Myodes glareolus* (5), *Microtus agrestis* (4), *Lemmus lemmus* (2), *Microtus oeconomus* (1), *Myodes rufocanus* (1), *Myodes rutilus* (2), *Myopus schistocolor* (3)	2011	68°01'46"N, 24°12'16"E
**3**	Harads (HA)	Sweden	*Myodes glareolus* (1)	2010	65°40'54"N, 21°30'16"E
**4**	Haparanda (HP)	Sweden	*Myodes glareolus* (1)	2011	65°48'47"N, 24°07'11"E
**5**	Fredrika (FE)	Sweden	*Myodes glareolus* (1)	2010	63°56'25"N, 17°25'56"E
**6**	Umeå (UM)	Sweden	*Myodes glareolus* (10)	2009-2012	63°53'22"N, 20°43'06"E
**7**	Gnarp (GN)	Sweden	*Myodes glareolus* (2)	2012	61°51'13"N, 17°12'08"E
**8**	Enånger (EN)	Sweden	*Myodes glareolus* (3)	2012	61°31'36"N, 17°01'11"E
**9**	Tierp (TI)	Sweden	*Myodes glareolus* (2)	2011	60°34'03"N, 17°48'40"E
**10**	Öster Malma	Sweden	*Myodes glareolus* (1)	2012	58°59'03"N, 17°05'07"E
**11**	Keava	Estonia	*Mus musculus* (1)	2012	58°56'27"N, 24°54'26"E
**12**	Nutter	The Netherlands	*Myodes glareolus* (1)	2012	52°24'46"N, 06°52'05"E
**13**	Jeeser	Germany	*Myodes glareolus* (1)	2010	54°09'25"N, 13°15'14"E
**14**	Billerbeck	Germany	*Myodes glareolus* (1)	2010	51°59'36"N, 07°19'01"E
**15**	Gotha	Germany	*Myodes glareolus* (2)	2010	50°57'17"N, 10°39'03"E
**16**	Weissach (WE)	Germany	*Myodes glareolus* (8)	2010	48°50'39"N, 08°57'25"E
**17**	Rozhanovce	Slovakia	*Myodes glareolus* (2)	2012	48°45'07"N, 21°20'37"E
**18**	Fugelka (FU)	Slovakia	*Myodes glareolus* (1)	2012	48°11'24"N, 07°04'57"E
**19**	La Venotiere (LA)	France	*Myodes glareolus* (2)	2014	47°44'25"N, 01°46'26"E
**20**	Vouzon	France	*Myodes glareolus* (3)	2014	47°39'34"N, 02°07'21"E
**21**	Mont-Sondrious-Vaudrey	France	*Myodes glareolus* (2)	2014	46°56'06"N, 05°33'45"E
**22**	Mignovillard (MI)	France	*Myodes glareolus* (3)	2014	46°46'32"N, 06°09'06"E
**23**	Cormaranche-en-Bugey	France	*Myodes glareolus* (1)	2014	45°56'00"N, 05°37'10"E
**24**	Brescia (BS)	Italy	*Myodes glareolus* (2), *Mus musculus* (1), *Sorex antinorii* (1)	2010 and 2012	46°13'16"N, 10°24'16"E
**25**	Pavia (PV)	Italy	*Sciurus vulgaris* (1)	2011	44°52'10"N, 09°08'05"E
**26**	Sondrio (SO)	Italy	*Myodes glareolus* (4), *Sorex antinorii* (1)	2008 and 2012	46°15'48"N, 10°16'39"E

**27**	Trento (TN)	Italy	*Myodes glareolus* (6), *Apodemus flavicollis* (1), *Microtus multiplex* (1)	2011-2012	45°58'39"N, 10°53'48"E
**28**	Lecco (LC)	Italy	*Sciurus vulgaris* (1)	2002	45°43'19"N, 09°26'36"E
**29**	Pula	Croatia	*Mus musculus* (1)	2012	44°50'45"N, 13°53'12"E

Supplementary Table S2. List of primers used to amplify LV 3D^{pol} and VP1 regions of LV. Primer position refers to the LV strain 87-012 genome sequence (GenBank acc. no.: AF327920).

Designation	Region amplified	Direction	Position	Sequence (5'→3')
PeV 3D F1*	3D^{pol}	forward	6602-6623	GNA ARA CYM GDT GYA THG ARG C
PeV 3D F2*	3D^{pol}	forward	6679-6695	THT ATG ANR MAA TYT AYC ARA CYC C
Lv 3D F1*	3D^{pol}	forward	6645-6668	GCC TAY AGA GTT GTD ATG TCN TC
Lv 3D F3^	3D^{pol}	forward	6772-6794	TAT AAY TAT GGY YTR GA TTA YTC
Lv 3D F4^	3D^{pol}	forward	7009-7028	TGT ACA TAT TTG GCC TAT GA
PeV 3D R1a*	3D^{pol}	reverse	7278-7303	TYT TCA TCC ACA TWA HRT GYT GTT CC
PeV 3D R1b*	3D^{pol}	reverse	7278-7303	TYT TCA TCC ACA TWA HRT GYT GTA TC
Pev 3D R2*	3D^{pol}	reverse	6967-6989	GAA TTY ARM ACA GTD GTR CAI GG
Lv 3D R1*	3D^{pol}	reverse	7278-7303	TYT TCA TCC ACA TAA TRT GYT GTT CC
Lv 3D R2*	3D^{pol}	reverse	7142-7163	TCT GAK GMN GTC ACT TCC ATN CC
Lv 3D R3^	3D^{pol}	reverse	6841-6863	TCA TGA CAR TAR GCA AGD ATT TC
Lv 3D R4^	3D^{pol}	reverse	7057-7079	ATY ACA TCA TYC CAT AAA CAA T
Lv VP1 F1^	VP1	forward	2284-2304	GAG ATW CAR GAY ADK GAR GAA
Lv VP1 F2*	VP1	forward	2659-2681	AAA GTT GCW CAY ACM TGG TTT GG
Lv VP1 F3^	VP1	forward	2773-2793	TAY TAY TCW GAR GYN CCA YT
Lv VP1 F4 I^	VP1	forward	2839-2863	TYT TYT AYC AGT GTG TTG GGC GCT
Lv VP1 F4 II^	VP1	forward	2839-2863	CTY TTY ACA CAR GCK GTR GGM AGR
Lv VP1 F4 III^	VP1	forward	2839-2863	TYT TYA CMC ART GYA TTG GMA AYA
Lv VP1 F5 I^	VP1	forward	2893-2913	AGY YTA CGC TGY CCY AAT TT
Lv VP1 F5 II^	VP1	forward	2893-2913	AGY TTY CGC TGT CCT AAT TT
Lv VP1 F5 III^	VP1	forward	2893-2913	AGY TTT CGC TGY CCM AAY TT
Lv VP1 R1*	VP1	reverse	3188-3208	TCR ATR TCR GGG CCW GGG TT
Lv VP1 R2a*	VP1	reverse	3341-3364	GCA TGT GTC CAA TTT CCA TCA TC
Lv VP1 R2b*	VP1	reverse	3341-3364	GCA TGA ACC CAT TTG CCA TCA TC
Lv VP1 R3^	VP1	reverse	3385-3404	TGT TTN GAR CCA ACC ATT GA
Lv VP1 R4 I^	VP1	reverse	3077-3102	TCY CTC AAT TGT GCC AAT GGR TCA T
Lv VP1 R4 II^	VP1	reverse	3341-3364	TCY CKM ART TGT TTC ARR GGR TCT T
Lv VP1 R4 III^	VP1	reverse	3341-3364	TCY CTM AGY TGT CTY ARR GGA TCH T
Lv VP1 R5 I^	VP1	reverse	3131-3156	CCA GAG TAT TCC ATT TCA TCA CTG T
Lv VP1 R5 II^	VP1	reverse	3131-3156	CCR GCA WAR TCC ATY TCA TCA CTR T
Lv VP1 R5 III^	VP1	reverse	3131-3156	CCR GAR TAR TCC ATY TCA TCA YTR T

* primers designed based on the deposited genomes of LV and HPeV.

^ additional primers designed based on the deposited genomes of LV and HPeV in combination with sequences obtained from this paper.

Supplementary Table S3. Samples used in the network analysis based on 5′-UTR sequences, including country and site of origin, host species and family/subfamily (subf.).

Country	Site*	Species (n°individuals)	Common name	Family
Finland	PJ	*Myodes glareolus* (4)	bank vole	Cricetidae - subf: Arvicolinae
Finland	PJ	*Microtus agrestis* (4)	field vole	Cricetidae - subf: Arvicolinae
Finland	PJ	*Lemmus lemmus* (2)	Norway lemming	Cricetidae - subf: Arvicolinae
Finland	PJ	*Myodes rutilus* (2)	northern red-backed vole	Cricetidae - subf: Arvicolinae
Finland	PJ	*Myopus schisticolor* (3)	wood lemming	Cricetidae - subf: Arvicolinae
Finland	KJ	*Lemmus lemmus* (3)	Norway lemming	Cricetidae - subf: Arvicolinae
Italy	SO	*Myodes glareolus* (23)	bank vole	Cricetidae - subf: Arvicolinae
Italy	SO	*Sorex antinorii* (1)	Valais shrew	Soricidae
Italy	TN	*Myodes glareolus* (4)	bank vole	Cricetidae - subf: Arvicolinae
Italy	TN	*Apodemus flavicollis* (4)	yellow-necked mouse	Muridae
Italy	BS	*Myodes glareolus* (2)	bank vole	Cricetidae - subf: Arvicolinae
Italy	BS	*Sorex antinorii* (1)	Valais shrew	Soricidae
Italy	BS	*Mus musculus* (3)	house mouse	Muridae
Italy	LC	*Sciurus vulgaris* (1)	Eurasian red squirrel	Sciuridae
Italy	PV	*Sciurus vulgaris* (1)	Eurasian red squirrel	Sciuridae

*For description and coordinates of sampling sites, see Table 1.
